# Plasmapheresis combined with rituximab treatment of a case of thrombotic thrombocytopenic purpura with Sjögren syndrome and renal impairment: A case report

**DOI:** 10.1097/MD.0000000000038103

**Published:** 2024-05-10

**Authors:** Yongqiang Zhang, Shanshan Hu, Yiyao Deng, Zhi Yang, Jing Yuan

**Affiliations:** a Guizhou University of Traditional Chinese Medicine, Guiyang 550002, Guizhou, China; b Guizhou Provincial People’s Hospital, Guiyang 550002, Guizhou, China.

**Keywords:** antibody, monoclonal, purpura, renal impairment, Sjögren syndrome, thrombotic microangiopathy, thrombotic thrombocytopenic

## Abstract

**Rationale:**

Thrombotic thrombocytopenic purpura (TTP) is a rare thrombotic microangiopathy caused by reduced activity of the von Willebrand factor-cleaving protease (ADAMTS13), which can be life-threatening. The patient reported in this case study also had concurrent Sjögren syndrome and renal impairment, presenting multiple symptoms and posing a great challenge in treatment.

**Patient concerns:**

A 25-year-old woman in the postpartum period visited the hospital due to indifference in consciousness for more than 1 day following cesarean section 8 days prior.

**Diagnosis:**

Notable decreases were observed in platelets, hemoglobin, creatinine, and ADAMTS13 levels. After a consultative examination by an ophthalmologist, she was diagnosed with retinal hemorrhage in the right eye and dry eye syndrome in both eyes.

**Interventions:**

Having been diagnosed with TTP with Sjögren syndrome and renal impairment, she received repeated treatments with plasmapheresis combined with rituximab.

**Outcomes:**

Following treatment and during the follow-up period, the patient’s platelet counts and bleeding symptoms significantly improved.

**Lessons:**

TTP has a high mortality rate, and when combined with Sjögren syndrome and renal impairment, it poses an even greater challenge in treatment. However, after administering standard plasmapheresis combined with rituximab treatment, the treatment outcome is favorable.

## 1. Introduction

Thrombotic thrombocytopenic purpura (TTP) is a rare thrombotic microangiopathy caused by a deficiency in the activity of ADAMTS13, a von Willebrand factor-cleaving protease. This leads to widespread microvascular thrombosis, causing microangiopathic hemolytic anemia, consumptive thrombocytopenia, and organ dysfunction in areas such as the heart, brain, and kidneys. The annual incidence of TTP is 2 to 6 per million people, with a female-to-male ratio of about 2:1, The disease typically peaks between the ages of 30 and 50. The majority of TTP patients have a rapid onset of symptoms and a critical condition,^[1,2]^ posing a serious threat to their lives. This report presents a case of TTP combined with Sjögren syndrome and renal involvement and discusses the efficacy of plasmapheresis combined with rituximab treatment for TTP combined with Sjögren syndrome and renal involvement through relevant literature.

## 2. Case information

The patient was a young woman, 25 years old, who was admitted to the emergency department of Guizhou Provincial People’s Hospital on February 8, 2022, due to “indifference in consciousness for more than 1 day following cesarean section 8 days prior.” Present Illness History: 8 days ago, the patient was full-term pregnant and had a platelet count of 21 × 10^9^/L at a local hospital. She was subsequently advised to transfer to our hospital’s obstetrics department for an “intraperitoneal cesarean section with transverse uterine incision + pelvic adhesion separation surgery.” The surgery went smoothly, with an estimated blood loss of about 400 mL. Postoperatively, her platelet count was 27.0 × 10^9^/L. However, she and her family refused further examination and treatment and insisted on signing out against medical advice. Three days prior, the patient experienced fatigue without an obvious cause, and a bruise appeared on the back of her left hand, approximately 6 × 3 cm² in size. Two days prior, she experienced a slight nosebleed, with no other symptoms such as gum bleeding, headache, dizziness, or blurred vision. She did not seek medical attention. One day prior, the patient developed indifference in consciousness without a clear trigger, accompanied by vomiting of gastric contents once and palpitations. There was no hematemesis, hematochezia, syncope, chest pain, limb spasms, or trismus. She sought emergency treatment at our hospital with “1. Suspected thrombotic thrombocytopenic purpura? 2. Severe hemolytic anemia? 3. Post cesarean section” and was admitted to the obstetrics department. Past medical history: she denied any history of infectious diseases such as hepatitis or tuberculosis, denied any history of cardiovascular diseases such as hypertension or coronary heart disease, denied any history of diabetes, cerebrovascular diseases, or mental illness, and vaccination history was unknown. She also denied any history of trauma or transfusion and any history of food or drug allergies. Upon admission, the physical examination revealed a temperature of 38.6°C, pulse of 127 beats/minute, respiration rate of 21 times/minute, blood pressure of 102/70 mm Hg, a 6 × 3-cm² bruise on the back of the left hand, and a 2 × 3-cm² bruise on the left buttock, with scattered petechiae on the skin. The abdominal wound was healing well; the abdomen was soft without tenderness, rebound tenderness, or muscle tension; the uterine fundus was 2 transverse fingers above the pubic symphysis, with scanty lochia without odor. Blood tests performed immediately after admission to obstetrics showed: white blood cells 16.69 × 10^9^/L, hemoglobin 54.0 g/L, platelets 4.0 × 10^9^/L, schistocytes 3%; coagulation tests showed: fibrin degradation products 16.1 μg/mL, D-dimer 5.31 μg/mL; antinuclear antibody spectrum showed: antinuclear antibody (Hep-2 cells + liver cells) - nuclear particle type +, antinuclear antibody titer 1:3200 +, anti-SS-A antibody positive (+++) RU/mL +, Ro-52 positive (+++) +, antiproliferating cell nuclear antigen antibody weakly positive RU/mL +. Abdominal gynecological ultrasound showed an enlarged uterus with no abnormalities of the appendages on both sides. Routine electrocardiogram showed: sinus tachycardia with a heart rate of 124 beats/minute and low voltage QRS waves in limb leads. Cranial and pulmonary CT scan showed pericardial effusion; lower density of the heart chamber and large blood vessels, suggesting anemia; no obvious abnormalities on cranial CT scan, consider MRI if necessary. As the patient’s platelet count was extremely low, which could lead to critical organ bleeding or spontaneous intracranial hemorrhage at any time, endangering her life, TTP could not be ruled out, prognosis was extremely poor, condition was critical, mortality was high, and treatment was difficult, so she was transferred to the ICU for continued treatment.

On February 8, 2022, at 21:11, the patient was admitted to the ICU. On examination, the patient had a temperature of 36.6°C, heart rate of 128 beats/minute, blood pressure of 111/67 mm Hg, respiratory rate of 25 breaths/minute, and an oxygen saturation level of 98% on nasal cannula oxygen. The patient appeared drowsy but was able to open eyes when called loudly. The patient was breathing spontaneously without obvious distress. There was a 6 × 3 cm² bruise on the back of the left hand and a 2 × 3-cm² bruise on the left buttock, with scattered petechiae on the skin. The pupils were round and equal in size, with brisk reaction to light. The breath sounds were clear in both lungs without significant dry or wet rales. The heart rhythm was regular. The abdomen was soft and non-tender. No enlargement of liver or spleen was palpable under the ribs, bowel sounds were weak, and there was no edema observed in both lower extremities. Repeat blood tests showed a platelet count of 4.0 × 10^9^/L, hemoglobin concentration of 49 g/L. High-sensitivity cardiac troponin I was 1.5420 μg/L, lactate dehydrogenase was 2091 U/L, and renal function showed a creatinine of 190 μmol/L with an estimated glomerular filtration rate of 31 mL/min/1.73 m². Liver function tests showed a total bilirubin of 36.2 μmol/L, direct bilirubin of 12.9 μmol/L, indirect bilirubin of 23.3 μmol/L, and albumin of 31.0 g/L. The erythrocyte sedimentation rate was 87 mm/h. After consultations with the departments of hematology, nephrology, rheumatology, and immunology, a strong possibility of TTP with renal failure was considered, and connective tissue diseases such as SLE or Sjögren syndrome were also suspected. Therefore, on February 9, 2022, intensive treatment was initiated: daily plasma exchange (2000 mL of fresh frozen plasma) to remove pathogenic antibodies, pulse therapy with methylprednisolone (1000 mg intravenous drip for 3 days, then gradually reduced to 40 mg daily to maintain), intravenous immunoglobulin (PH4) at 20 g daily for 5 days to block pathogenic antibodies, rituximab at 375 mg/m² intravenous drip weekly to induce apoptosis of B cells, and blood transfusions (washed red blood cells 2 units intermittently) to improve anemia. During the treatment, ADAMTS13 activity was tested, revealing inhibitory antibodies (+) and ADAMTS13 activity at 2.15%, which confirmed the diagnosis of TTP. Additional tests including antineutrophil cytoplasmic antibody spectrum, autoimmune hepatitis antibody spectrum, antistreptolysin O, anticyclic citrullinated peptide antibody, CD55, CD59, direct antiglobulin test, acid hemolysis test, infectious disease screening, and tuberculosis T-SPOT did not show any remarkable abnormalities. Following the intensive treatment, on February 16, 2022, a repeat complete blood count showed hemoglobin of 82.0 g/L and platelets of 264.0 × 10^9^/L. The anemia and thrombocytopenia showed significant improvement, and the patient’s mental state became clearer. To further clarify the causes of connective tissue disease and renal failure, the patient was transferred to the Nephrology, Rheumatology, Immunology Department for further treatment on February 16, 2022. Up until this point, the patient had received 2 days of 1000 mg daily methylprednisolone, 3 days of 80 mg daily, 2 days of 40 mg daily, had undergone 4 plasma exchange sessions, and had received 2 doses of rituximab at 600 mg intravenous drip weekly according to the treatment plan.

**Figure 1.**
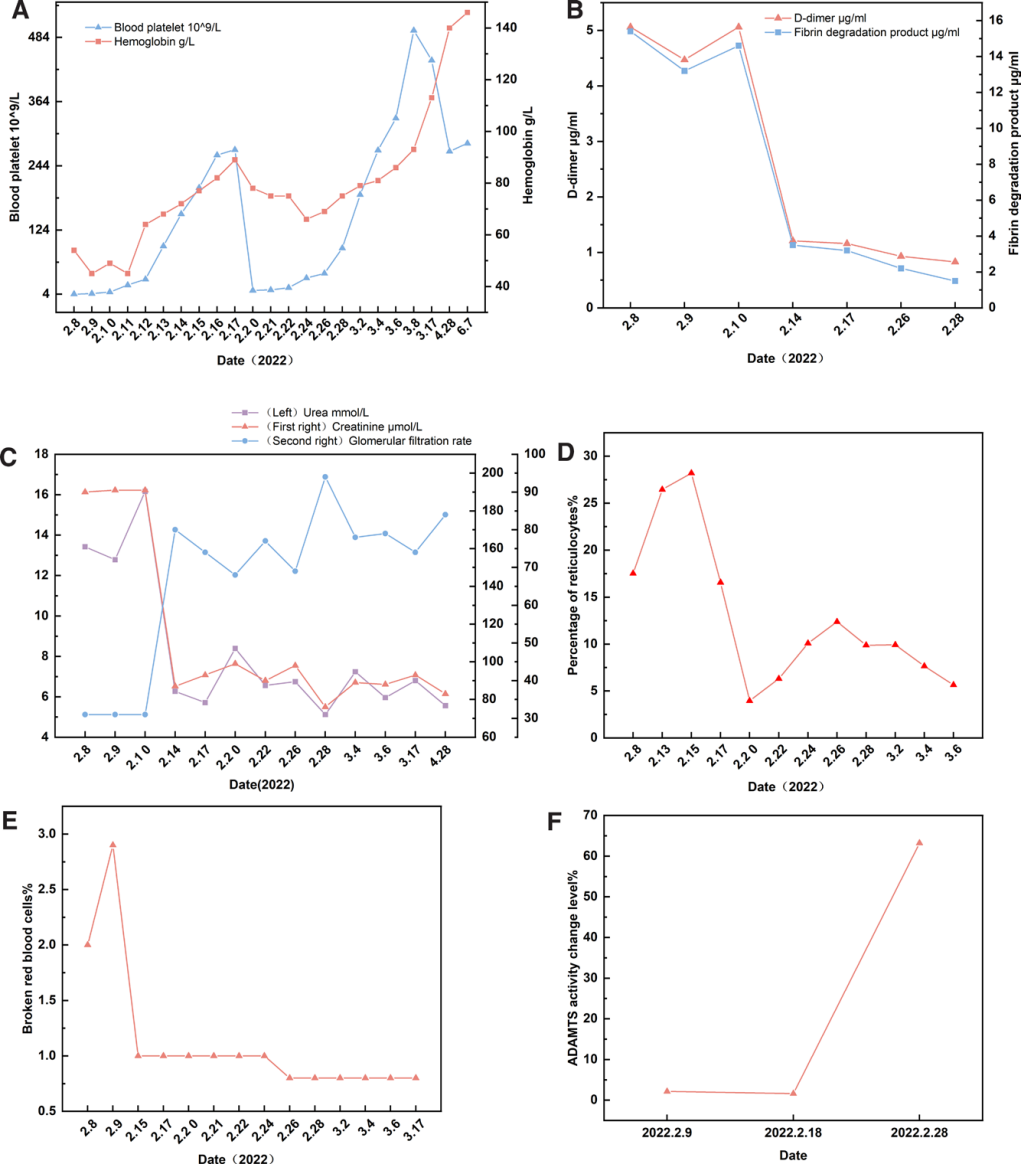
Changes in the patient’s laboratory test indices. (A) Levels of platelet and hemoglobin changes. (B) Change level of coagulation function. (C) Levels of changes in renal function-related indicators (urea, creatinine, glomerular filtration rate). (D) Change level of reticulocyte percentage. (E) Change level of broken red blood cells. (F) ADAMTS activity change level.

After being transferred to the Nephrology, Rheumatology, Immunology Department, further tests were conducted. Complete blood count: hemoglobin: 89.0 g/L; platelets: 274.0 × 10^9^/L; erythrocyte sedimentation rate: 21 mm/h; immunoglobulin G: 20.2 g/L. Other tests including routine urinalysis, complements C3, C4, and C1q, C-reactive protein, tumor markers, schistocytes (fragmented red blood cells), procalcitonin, coagulation profile, serum protein electrophoresis, and lupus anticoagulant were all within normal ranges. Ophthalmology consultation: Right eye: dot-and-blot hemorrhages noted adjacent to optic disc. Tear break-up time: 5 seconds for the right eye, 8 seconds for the left eye. Schirmer test (tear secretion test): 6 mm for the right eye, 4 mm for the left eye. Diagnosis: Hemorrhage in the retina of the right eye, dry eye syndrome in both eyes. The differential diagnosis considered are TTP; connective tissue disease – Sjögren syndrome, systemic lupus erythematosus (SLE); and underlying cause of renal insufficiency remains to be investigated. On February 20, 2022, a repeat CBC revealed a sharp decline in platelets to 11.0 × 10^9^/L, which was thought to be associated with uncontrolled TTP. The patient was then given a 3-day course of pulse therapy with methylprednisolone (200, 300, and 300 mg), IV immunoglobulin at 20 g daily for 3 days, along with plasma exchange (2000 mL of fresh frozen plasma) every other day. Hydroxychloroquine sulfate was also administered orally at a dose of 0.2 g twice a day to modulate the immune system. On February 24, a follow-up revealed platelets at 34.0 × 10^9^/L, 1% schistocytes, ADAMTS13 activity at 1.63%, and negative ADAMTS13 inhibitory antibodies, suggesting that TTP was not completely controlled. A reduced immune-related platelet count was also not excluded; thus, cyclosporine capsules at 50 mg were added orally twice a day as immune suppression. Due to fluctuating platelet counts in the past few days, plasma exchanges were continuously conducted from February 26 to 28, using 2000 mL of fresh frozen plasma each day. On the same days, hemoglobin was measured at 75.0 g/L and platelets at 90.0 × 10^9^/L. For the consolidation of the treatment’s effect, plasma exchanges were continued on March 1 and 2, and results showed hemoglobin at 79.0 g/L and platelets at 190.0 × 10^9^/L with schistocytes < 1%. On March 3 and 4, the therapy persisted with plasma exchanges, and on March 4, tests revealed ADAMTS13 activity at 63.22%, negative ADAMTS13 inhibitory antibodies, hemoglobin at 81.0 g/L, and platelets at 273.0 × 10^9^/L, with schistocytes < 1%. In accordance with the therapeutic regimen, a final dose of rituximab injection at 600 mg was administered for immune suppression. On March 8, the patient’s hemoglobin level improved to 93.0 g/L and platelets to 497.0 × 10^9^/L. The patient’s condition became relatively stable, and they were discharged with advice to maintain a low-salt, low-fat diet, rest sufficiently, avoid cold exposure, enhance nutrition, and not to stop or reduce medication on their own. Follow-up visits were scheduled to monitor CBC, liver and kidney function, antinuclear antibody spectrum, and other relevant indicators. After discharge, the patient revisited the hospital for follow-up on March 17, April 28, and June 7. The patient exhibited significant improvements in platelets, hemoglobin, and kidney function (Fig. 1A and B).

## 3. Outcomes

From February 8, 2022, to February 16, 2022, the patient received methylprednisolone 1000 mg QD for 2 days, 80 mg QD for 3 days, and 40 mg QD for 2 days, plasma exchange for 4 times, and rituximab 600 mg intravenous QW was used for 2 times according to the treatment course. At the same time, from February 16, 2022, to March 4, 2022, the patient received multiple plasma exchanges combined with rituximab treatment in the Department of Renal Rheumatology and Immunology. After treatment, the platelet and hemoglobin increased significantly (Fig. 1A), D-dimer and fibrin degradation products decreased significantly (Fig. 1B), renal function improved significantly (Fig. 1C), reticulocyte percentage improved (Fig. 1D), broken red cell count decreased (Fig. 1E), and ADAMTS13 increased significantly (Fig. 1F).

## 4. Discussion

TTP is a rare and severe microvascular thrombotic disease,^[3]^ primarily caused by a severe deficiency of ADAMTS13. The deficiency or decreased activity of ADAMTS13 leads to the formation of excessive ultra-large von Willebrand factor multimers in the blood, resulting in endothelial damage. This can trigger the aggregation of platelets and further lead to the formation of microthrombi, causing TTP. Based on the mechanism of ADAMTS13 deficiency, TTP is classified into hereditary TTP (congenital TTP, also known as Upshaw–Schulman syndrome) and immune-mediated TTP. Although the incidence of TTP is relatively low, most patients have a rapid onset and critical illness. Without prompt recognition and treatment, the mortality rate can be as high as 90%.^[4]^ Some patients may ultimately progress to end-stage renal disease.^[5]^

The main clinical manifestations of TTP include the classic “pentad” of fever, microangiopathic hemolytic anemia (MAHA), thrombocytopenia, renal dysfunction, and neurological symptoms. However, this “pentad” is not often seen clinically; a “triad” of MAHA, thrombocytopenia, and neuropsychiatric symptoms is more common. The “2022 Chinese Guidelines on the Diagnosis and Treatment of Thrombotic Thrombocytopenic Purpura” state that the laboratory diagnosis includes varying degrees of anemia, fragmented red blood cells (>1%) seen on peripheral blood smear, with most cases showing an increased proportion of reticulocytes. Platelet counts are significantly reduced (often lower than 20 × 10^9^/L), with a marked dynamic decrease, and plasma ADAMTS13 activity is significantly reduced (<10%). In this case, the patient’s initial hemoglobin was 54.0 g/L, platelets were 4.0 × 10^9^/L, fragmented red blood cells were 3%, reticulated red blood cells percentage was 17.51%, and the patient had positive inhibitory antibodies to ADAMTS13 with an ADAMTS13 activity level of 2.15%, thus confirming the diagnosis of TTP. Additionally, the guidelines recommend plasma exchange as the first-line treatment to remove ADAMTS13 inhibitors or IgG antibodies and other pathogenic factors, optionally combined with corticosteroids. Rituximab, a humanized anti-CD20 monoclonal antibody, may reduce the production of autoantibodies by decreasing B lymphocyte numbers and is increasingly used in early treatment, improving disease remission and disease-free intervals. When plasma exchange and corticosteroids alone are not effective, combining treatment with rituximab is a safe and effective method to prevent acute relapse.^[6,7]^

After treatment with intravenous immunoglobulin, steroid pulse therapy, and red blood cell transfusion, although the patient’s hemoglobin level increased, it did not return to normal, and the platelet count showed no significant change, remaining low and increasing the risk of visceral and intracranial bleeding. The presence of a positive antinuclear antibody spectrum suggested immune-related thrombocytopenia, possibly related to connective tissue disease. Following 4 consecutive days of plasma exchange, the platelet count rose to 154.0 × 10^9^/L, indicating that the combination of plasma exchange, immunoglobulin, and steroid pulse therapy can be effective for acute phase patients. However, 3 days later, a retest showed that the platelet count had dropped to 11.0 × 10^9^/L, suggesting that the volume of plasma exchange was insufficient and had not completely removed autoantibodies. Therefore, after an additional ten plasma exchanges, the platelets increased to 497.0 × 10^9^/L. Upon follow-up on June 7, 2022, the candidate’s platelet count was stable at 497.0 × 10^9^/L. This case suggests that for TTP combined with autoimmune connective tissue diseases, adopting plasma exchange in conjunction with biologics and adequate doses and courses of steroid pulse therapy provides a new approach. The definitive efficacy of this treatment strategy requires validation through further clinical trials.

## 5. Limitations

During the hospital stay, the patient was positive for antinuclear antibodies and lupus anticoagulant tests: lupus anticoagulant screening at 27.7 seconds, and lupus coagulant confirmation at 28.1 second. Due to the patient’s poor platelet and coagulation functions, the risk associated with biopsy was increased, and therefore, bone marrow aspiration, renal biopsy, and lip gland biopsy were not performed. This study is only a case report, more clinical application evidence is required, which calls for long-term observations and evaluations in large-sample case studies.

## Author contributions

**Data curation:** Yongqiang Zhang, Zhi Yang.

**Visualization:** Yongqiang Zhang, Zhi Yang.

**Writing – original draft:** Yongqiang Zhang.

**Supervision:** Shanshan Hu, Yiyao Deng.

**Writing – review & editing:** Shanshan Hu, Jing Yuan.

**Resources:** Yiyao Deng, Jing Yuan.

**Investigation:** Jing Yuan.
